# Prevalence of COVID-19 in Kidney Transplant Patients in Relation to Their Immune Status after Repeated Anti-SARS-CoV-2 Vaccination

**DOI:** 10.3390/pathogens12020351

**Published:** 2023-02-19

**Authors:** Sandra Sakalauskaite, Ruta Vaiciuniene, Neda Kusleikaite-Pere, Jurgita Narbutiene, Jolanta Sauseriene, Asta Aukstakalniene, Leonas Valius, Brigita Sitkauskiene

**Affiliations:** 1 Laboratory of Immunology of the Department of Immunology and Allergology, Lithuanian University of Health Sciences, LT-50161 Kaunas, Lithuania; 2 Department of Nephrology, Lithuanian University of Health Sciences, LT-50161 Kaunas, Lithuania; 3 Department of Family Medicine, Lithuanian University of Health Sciences, LT-50161 Kaunas, Lithuania; 4 Department of Immunology and Allergology, Lithuanian University of Health Sciences, LT-50161 Kaunas, Lithuania

**Keywords:** anti-SARS-CoV-2, COVID-19, immune response, kidney transplant, vaccination

## Abstract

The prospective study was conducted to evaluate the prevalence of COVID-19 in kidney transplant patients in relation to their immune status after three doses of the BNT162b2 (Pfizer-BioNTech) vaccine during one post-pandemic year based on the experience of one center—Hospital of Lithuanian University of Health Sciences. Thirty-three patients were invited for a follow-up visit 3 to 6 weeks after anti-SARS-CoV-2 vaccination and were obliged to report having COVID-19 during the one-year post-pandemic period. Forty-two percent of patients developed antibody response against SARS-CoV-2 after the third dose of the vaccine. The number of COVID-19 cases during the post-pandemic period did not differ significantly between seropositive and seronegative patients. However, only seronegative patients were hospitalized due to COVID-19. The anti-SARS-CoV-2 antibody titer in seropositive patients correlated with a relative number of CD3^+^ cells (R = 0.685, *p* = 0.029). The CD8^+^/CD38^+^ ratio in this group increased 2-fold after the anti-SARS-CoV-2 vaccination. Higher antibody response to the COVID-19 vaccine was associated with better kidney function. The anti-SARS-CoV-2 antibody titer relation with the components of cellular immunity (CD3^+^ cells and CD8^+^/CD38^+^ ratio) shows a role of both chains during the response to the anti-SARS-CoV-2 vaccine in kidney transplant patients.

## 1. Introduction

Coronavirus disease 2019 (COVID-19) is a highly contagious viral disease caused by severe acute respiratory syndrome coronavirus 2 (SARS-CoV-2). Despite significant clinical trials and progress in managing COVID-19, the emergence of new strains remains a concern. Elucidating the mechanisms of the immune response, especially in patients with immunosuppressive risk factors, would provide knowledge about limiting the spread of this disease and the risk of hospitalizations and deaths. Previous studies showed that COVID-2019 significantly affects kidney and other solid organ transplant recipients [1]. Vaccination has become a key tool for controlling the ongoing pandemic.

Since the SARS-CoV-2 virus emerged, great attention has been paid to the formation of the immune response. The SARS-CoV-2 infection affects the stimulatory levels of cellular-mediated immunity, which is essential in controlling SARS-CoV-2 infection [2]. Data show that during the COVID-19 infection, T and B cells work in concert alongside the instructive innate immune system to control the virus, displaying distinct response kinetics, mode of antigen recognition, effector functions, and immunological memory as in other viral infections. Typically, B cells produce antibodies responding to pathogens or vaccination and act as the first line of defense. T cells have an additional important role in containing the infection through their ability to eliminate already infected cells. Meanwhile, CD4^+^ helper T cells provide signals that support the development of antibody responses and other functions [3]. However, the immune response is suboptimal in patients with chronic diseases (such as hypertension and cardiovascular disease) and those receiving immunosuppressive therapy, such as kidney transplant patients or patients requiring dialysis [4,5,6]. Anti-SARS-CoV-2 vaccine data showed that seroconversion rates remained reduced in immunocompromised groups (organ transplant recipients, patients with solid or hematological cancers, or patients with immune-mediated inflammatory disorders) compared with immunocompetent controls [7]. Therefore, patients with kidney diseases are at a high exposure of developing COVID-19, and the risk of death is about 25% in this group [8]. The phase II-III trial and real-world data revealed that the first vaccine BNT162b2 (Pfizer-BioNTech) showed 95% efficiency in preventing COVID-19 and hospitalization or death associated with SARS-CoV-2 infection in healthy adults [9]. The results of studies with kidney transplant patients vary, but the average vaccination efficiency after three doses reaches 48% [10,11,12]. However, therapeutic immunosuppression impairs responses to the mRNA anti-SARS-CoV-2 vaccine in kidney transplant patients; therefore, the COVID-19 vaccination program requires a personalized plan for these patients.

The immune system’s role in the pathophysiology of SARS-CoV-2 infection and during vaccination against COVID-19 is inconclusive. Risk factors associated with unfavorable outcomes were identified, including elderly age, selected comorbidities, immune suppression, and laboratory markers [13]. Despite much research being done, the question remains whether to continue vaccination with booster doses of the anti-SARS-CoV-2 vaccine, and further recommendations about the response to those vaccines are needed. In addition, there needs to be a definition of an adequate response or correlate of morbidity after the third dose of the anti-SARS-CoV-2 vaccine in kidney transplant patients. Furthermore, restrictions during the pandemic years reduced the intensity of social life, so the results of the pandemic period on the morbidity of vaccinated individuals may be biased. We aimed to evaluate the prevalence of COVID-19 in kidney transplant patients after repeated anti-SARS-CoV-2 vaccination during the last year, when pandemic restrictions were lifted and people returned to their normal rhythm of life. This study tended to improve our understanding of the immune response in kidney transplant patients after the three doses of the mRNA anti-SARS-CoV-2 vaccine (BNT16B2) and the relation with COVID-19 morbidity during one post-pandemic year after the vaccination based on the experience of one center—Hospital of Lithuanian University of Health Sciences.

## 2. Materials and Methods

This prospective non-randomized study was conducted in the Hospital of Lithuanian University of Health Sciences, Kaunas clinics. The permission was issued by the Regional Bioethical Committee on 5 February 2021 (No. BE-2-43). Data for this study were collected between September 2021 and October 2022.

Thirty-three kidney transplant patients (20 men and 13 women) were vaccinated with three doses of BNT162b2 (Pfizer-BioNTech) SARS-CoV-2 mRNA vaccine. None of the patients had COVID-19 previously or between vaccinations. The first two doses of vaccine were given as recommended by the manufacturer (at an interval of 21 days). The 3rd vaccination was applied after 8 months. Patients came for a follow-up visit 4 to 6 weeks after immunization (mean time 54± 24 days). Patients’ medical records were used to collect data about comorbid conditions and medications. Anamnesis of angina pectoris, myocardial infarction, and stroke were considered cardiovascular events. Indicators of kidney function (total level of creatinine and estimated glomerular filtration rate (eGFR, calculated by CKD-EPI formula)), hemoglobin (Hb), and lymphocyte subpopulations were evaluated before and 4–6 weeks after anti-SARS-CoV-2 vaccination. Blood samples were taken to assess anti-SARS-CoV-2 spike-specific IgG class antibodies and lymphocyte subpopulations four to six weeks after vaccination. The study subjects were obliged to report having COVID-19 during the one-year post-pandemic period (September 2021 to October 2022) after the 3rd dose of the vaccine. During the study, physicians followed their patients regarding COVID-19 cases by patients’ self-declaration and medical records using a unified e-system.

### 2.1. Measurement of Anti-SARS-CoV-2 Spike Protein IgG Antibodies

QuantiVac ELISA assay (Euroimmun) was used for quantitative in vitro determination of human antibodies of the IgG anti-SARS-CoV-2 spike proteins in serum. The research was carried out in accordance with the manufacturer’s recommendations. Values were given in BAU/mL (BAU—binding antibody units). When the result was >35.2 BAU/mL, the subject was defined as “seropositive”.

### 2.2. Evaluation of Lymphocyte Subpopulation

Flow cytometry (BD FACSLyric, BD Biosciences, San Diego, CA, USA) was used to quantify lymphocyte subpopulations in blood serum. After blood incubation with a monoclonal antibody mix (BD Multitest 6-color TBNK reagent, BD Biosciences, San Diego, CA, USA) and erythrocyte lysis, samples prepared following manufacturer guidance were acquired and analyzed on the BD FACSLyric system with BD FACSuite Clinical software (BD Biosciences, San Diego, CA, USA). The lymphocyte region was gated during data analysis, and the absolute numbers (cells/L) of lymphocyte subpopulations in the sample were established. T, B, and NK cells were characterized by the expression of specific CD markers.

### 2.3. Statistical Methods

Statistical analysis was performed using Statistical Package for Social Science (SPSS) version 22.0 (IBM SPSS Statistics for Microsoft Windows, Version 22.0. Armonk, NY: IBM Corp). In relation to data distribution, continuous variables have been described as mean with standard deviation or median. According to the level of anti-SARS-CoV-2 spike protein antibodies, patients were defined as “seropositive” (anti-SARS-CoV-2 ≥ 35.2 BAU/mL) or “seronegative” (anti-SARS-CoV-2 < 35.2 BAU/mL). The Kolmogorov–Smirnov test was applied to check quantitative variables for Gaussian distribution. The seropositive and seronegative patient group results were compared by analyzing means of an independent *t*-test or the one-way ANOVA test. Ordinal variables were analyzed using the Kendal tau-b test. Spearman’s coefficient was calculated for testing bivariate correlations of antibody titers with other variables. Changes in the variables before and after anti-SARS-CoV-2 vaccination were evaluated by paired *t*-test and Wilcoxon signed-rank test for related samples. *p*-values below 0.05 were considered statistically significant for all analyses.

## 3. Results

### 3.1. The Anti-SARS-CoV-2 Antibody Titer and COVID-19 Cases

**Figure 1:**
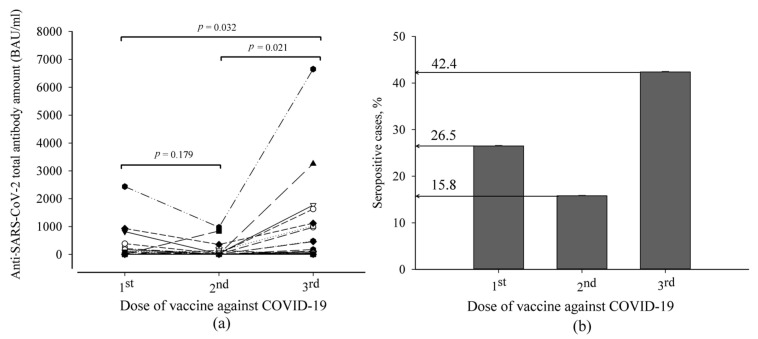
The distribution of anti-SARS-CoV-2 antibody titer in kidney transplant patients after anti-SARS-CoV-2 vaccination: (**a**) the anti-SARS-CoV-2 variation curve of an individual patient after each dose of vaccine; (**b**) percentage of seropositive cases from all studied subjects.

In this prospective non-randomized study, the median age [SD] of recruited kidney transplant patients (20 men and 13 women who were at least three months post-transplant) was 58 [11.85] (26–70) years. Some of them had comorbidities: 22.6% had diabetes and cardiovascular diseases (including hypertension), and 16.1% had oncological diseases. Coexisting diseases did not impact the titer of anti-SARS-CoV-2 spike protein IgG antibodies. The total amount of anti-SARS-CoV-2 antibodies in kidney transplant patients was tested after each dose of the vaccine. Their changes are shown in Figure 1. The means of total antibody amount between the first and second doses of the anti-SARS-CoV-2 vaccine had no significant difference. A significant difference appeared only after the booster with the third dose. After the first dose of the vaccine against COVID-19, 26.5% of patients developed an antibody response (>35.2 BAU/mL) against SARS-CoV-2. After the second dose, this value decreased to 15.8%. The third dose of the vaccine increased the seropositive cases by 2.7 times compared to the second dose. Furthermore, there was a positive correlation between specific antibody titer after the first dose of vaccination and that after the third dose (Spearman’s correlation coefficient 0.576, *p* = 0.001), which means that for those patients who responded after the first dose, antibody concentrations remained higher after the third dose.

The association of morbidity with the data of specific antibodies and immune markers developed during the year after the third dose of the vaccine was evaluated. There was no significant difference between the numbers of COVID-19 cases in seronegative and seropositive patients (47% and 46%, respectively) which showed a high rate of symptomatic disease. The symptoms were mild in most cases. However, crosstabulation analysis showed that 21% of these patients were hospitalized, and all of them were seronegative (*p* < 0.025). Therefore, the anti-SARS-CoV-2 antibody amount did not correlate with COVID-19 morbidity (Spearman’s correlation coefficient 0.142, *p* = 0.439) or hospitalization (Spearman’s correlation coefficient 0.289, *p* = 0.103) in studied subjects. Diabetes and cardiovascular diseases (including hypertension) could not be identified as risk factors for a more severe course of COVID-19 (Kendall’s tau-b correlation coefficients: r = −0.323 and −0.164, *p* = 0.082 and 0.376, respectively). A negative correlation was observed between oncological diseases and COVID-19 morbidity (Kendall’s tau-b correlation coefficient r = −0.391, *p* 0.035).

Two groups of patients (seronegative and seropositive) were compared to identify the related factors that may influence the immune response of kidney transplant patients after anti-SARS-CoV-2 vaccination. (Table 1).

### 3.2. Formation of Immune Response, Kidney Function, and Other Factors

**Figure 2:**
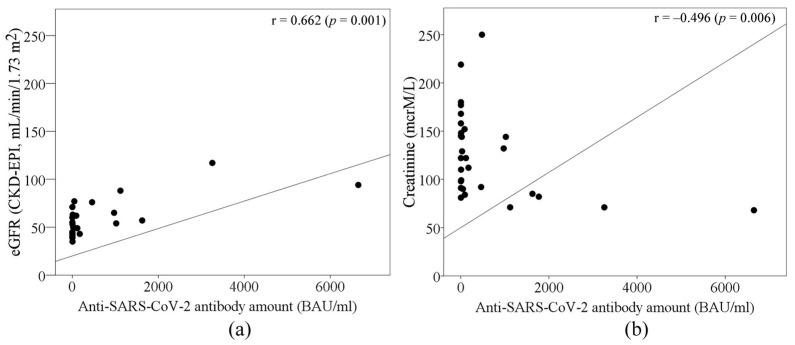
The role of (**a**) eGFR and (**b**) blood creatinine levels in antibody response after the 3rd anti-SARS-CoV-2 vaccination in kidney transplant patients.

Out of all measures, the estimated glomerular filtration rate (eGFR) and blood creatinine before and after the third dose of the anti-SARS-CoV-2 vaccine differed significantly between the groups according to their seropositivity. SARS-CoV-2 seropositive subjects had higher eGFR levels and lower blood creatinine levels (Table 1). Moreover, the results showed that the eGFR rate moderately correlated with the anti-SARS-CoV-2 antibody amount after the third dose of the vaccine (r = 0.662, *p* < 0.05). Meanwhile, a negative correlation between anti-SARS-CoV-2 antibody amount and blood creatinine level (r = 0.496, *p* < 0.05) was determined (Figure 2). A strong positive correlation was determined between eGFR and activated CD3^+^/HLA cells in all studied subjects (Spearman’s correlation coefficient 0.772, *p* = 0.042). After separating seronegative and seropositive cases, eGFR and activated CD8^+^/CD38^+^ cells were found to exhibit a strong negative correlation in seronegative patients and a strong positive correlation in seropositive patients (Spearman’s correlation coefficients −1 and 1, respectively, *p* < 0.001). A moderate negative correlation between blood creatinine levels and activated CD3^+^/HLA cells was determined in all studied groups (Spearman’s correlation coefficient −0.655, *p* = 0.015). A strong negative correlation was observed between blood creatinine levels and activated CD8^+^/CD38^+^ cells in the seropositive group (Spearman’s correlation coefficient −0.9, *p* = 0.037); in the seronegative group, these parameters did not correlate.

**Figure 3:**
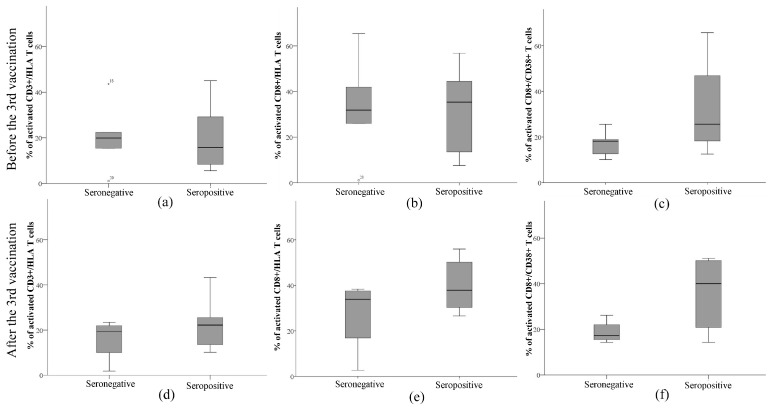
Evaluation of the number of activated immune cells, namely (**a**,**d**) CD3^+^, (**b**,**e**) CD8^+^, and (**c**,**f**) CD8^+^/CD38^+^, (**a**–**c**) before and (**d**–**f**) after the third dose of anti-SARS-CoV-2 vaccine in kidney transplant patients.

A deeper analysis of immune indicators in seronegative and seropositive patients before and after the third dose of the anti-SARS-CoV-2 vaccine was performed (Table 2, Figure 3). The results showed no significant differences between the means of CD3^+^ and CD8^+^ T cells in the seronegative and seropositive groups. On the other hand, the amount of CD8^+^ cells after vaccination correlated with anti-SARS-CoV-2 antibody titer in the seronegative group (Spearman’s coefficient r = 0.949, *p* = 0.05). The median counts of CD3^+^ and CD8^+^ T cells after vaccination slightly decreased in the seronegative group and increased in the seropositive group (Table 2).

Additional evaluation of the numbers of activated immune components, namely CD3^+^/HLA, CD8^+^/HLA, and CD8^+^/CD38^+^, is presented in Figure 3. The results showed that seropositive subjects had a lower median value of activated CD3^+^/HLA cells than seronegative ones. Furthermore, there was a positive correlation between CD3^+^/HLA and anti-SARS-CoV-2 antibody titer in the seropositive group (Spearman’s correlation coefficient 0.685, *p* = 0.029). After the third dose of the anti-SARS-CoV-2 vaccine, this value increased by 7% and was higher than that in seronegative patients. The median count of activated CD3+/HLA cells did not change after the repeated vaccination. Seropositive patients had a slightly higher median count of activated CD8^+^/HLA cells than seronegative patients. The median values were almost unchanged after the vaccination in both groups. The most striking difference between the median values of seronegative and seropositive subjects was recorded in the assessment of the amount of CD8^+^/CD38^+^. The median of the CD8^+^/CD38^+^ ratio was slightly higher in seropositive patients before the vaccination and increased 2 times after the vaccination, while it did not change in the seronegative patients. Moreover, activated CD8^+^/CD38^+^ negatively correlated with anti-SARS-CoV-2 antibody titer in this group (Spearman’s correlation coefficient −0.949, *p* = 0.05). The means of immune cell counts before and after vaccination were not significantly different within or between groups and did not relate to the prevalence of COVID-19.

## 4. Discussion

This study aimed to increase knowledge about the immune status in kidney transplant patients after three doses of a vaccine against COVID-19 and the relation with COVID-19 morbidity during a one-year post-pandemic period after vaccination based on the experience of one center—Hospital of Lithuanian University of Health Sciences. Key findings of this study were as follows: Measurable antibody response was detected in almost half of the patients (42.4%) after the third dose of the anti-SARS-CoV-2 vaccine. The level of antibodies did not correlate with the number of COVID-19 cases; however, only patients who did not develop a sufficient antibody response after repeated vaccination experienced hospitalization due to COVID-19. Analysis of immune response in kidney transplant patients showed that the antibody titers after repeated anti-SARS-CoV-2 vaccination positively correlated with the relative number of CD3^+^ cells. Antibody response and the amount of activated CD3^+^/HLA and CD8^+^/CD38^+^ cells are related to kidney function in these patients.

Publications issued during the pandemic period have assessed COVID-19 morbidity during social isolation. In contrast, our study was constructed to reflect a real-life situation and to present the incidence of COVID-19 after the third dose of the anti-SARS-CoV-2 vaccine at the end of pandemic restrictions. This study was focused on kidney transplant patients from a single study center. The results showed that anti-SARS-CoV-2 spike protein antibody titer significantly increased only after the third dose of the COVID-19 vaccine in these patients. After the first dose of the COVID-19 vaccine, 26.5% of studied patients developed an antibody response against SARS-CoV-2. After the second dose, this value decreased to 15.8%. The third dose of the vaccine increased the percentage of seropositive cases to 42.4. For those patients who responded after the first dose, antibody concentrations remained higher after the third dose. Such results suggest that an additional dose of the vaccine leads to a more robust immune response in kidney transplant patients despite their primary reaction to the vaccine or comorbidities. Consistency of response to an additional dose of the vaccine was observed in the work of Thomson et al. [14]. Those patients who had lower responses to the third and fourth doses of the vaccine had lower antibody titers after the second dose. Another randomized study found that 39% of kidney transplant patients without an immune response against SARS-CoV-2 after two mRNA vaccines developed antibodies against the SARS-CoV-2 spike protein four weeks after a third dose of an mRNA vaccine [15]. Although levels of antibodies against SARS-CoV-2 may contribute to infection clearance [16], we did not determine a significant difference in COVID-19 morbidity during a post-pandemic period between seronegative and seropositive kidney transplant patients (47% and 46%, respectively), which showed a high rate of symptomatic disease. Furthermore, the symptoms were mild in most cases and similar to those indicated in non-transplant subjects. However, only seronegative patients (with low antibody response after repeated anti-SARS-CoV-2 vaccination) experienced hospitalization due to COVID-19; these data show that they had more severe COVID-19 cases than seropositive patients with kidney transplants.

On the other hand, protection against viruses is primarily provided by cellular immunity. Various studies have shown that cell-mediated immunity, generally CD8+, is essential in eliminating virus-infected cells during severe infections [17]. Furthermore, it was determined that the levels of CD3^+^ and CD8^+^ T cells were significantly reduced in moderate COVID-19 and under-medication groups compared to a healthy group [2,18]. A previous study with healthy subjects vaccinated against COVID-19 also revealed that CD3^+^ and CD8^+^ T cells are essential vaccine-induced effector cells [19]. These cells may be the main mediators in the early stage of protection after primary vaccination, precede the maturation of other effector arms of vaccine-induced immunity, and are stably maintained after boost vaccination [19,20]. However, data on the cellular response after the COVID-19 vaccine in kidney transplant patients are limited. Our findings in the current study showed that kidney transplant patients who did not develop an antibody response after anti-SARS-CoV-2 vaccination had lower amounts of CD3^+^ and CD8^+^ T cells. Moreover, these amounts did not change after repeated vaccination. On the contrary, the median amount of CD3^+^ and CD8^+^ T cells increased in seropositive patients and was related to the anti-SARS-CoV-2 antibody titer. Despite that, there was no statistically significant difference between the mean amounts of these cells before and after vaccination. There are data showing that low counts of CD3^+^ and CD8^+^ T cells were associated with the risk of hospitalization in kidney transplant patients [8]. However, some studies showed that CD8^+^ T cells may increase immediately after the first two doses of the vaccine when antibodies have not yet been formed in kidney transplant patients [20,21]. Meanwhile, antibody levels increase after an additional booster (third) dose [19,22]. Therefore, data from our study and others show that CD8^+^ and CD3^+^ T cells may play an important role in maintaining immune function and viral clearance in the body during COVID-19 infections in kidney transplant patients.

Our study showed that the capability of response to the anti-SARS-CoV-2 vaccine in kidney transplant patients is related to their kidney function. We found that eGFR positively correlated with activated CD3^+^/HLA and CD8^+^/CD38^+^ cells. At the same time, high creatinine levels resulted in low levels of activated CD3^+^/HLA and CD8^+^/CD38^+^ cells. Such results indicate that renal function is important for the immune response forming after anti-SARS-CoV-2 vaccination in kidney transplant patients. Our statements are in line with those of other researchers. eGFR could be one of the most essential factors predicting survival after COVID-19 infection in kidney transplant patients [23]. The findings state that a decrease in eGFR could be associated with impaired cellular response to the COVID-19 vaccine in kidney transplant patients [24]. Moreover, low kidney function has been shown to impair the innate and adaptive immune response due to decreased B and T lymphocyte counts and poor lymphocyte activation, impaired monocyte function, inadequate antigen presentation, weakened memory cell generation, and low antibody production [25].

While elucidating the determinants of the immune response of kidney transplant patients after repeated anti-SARS-CoV-2 vaccination, a lack of correlation was observed between comorbidities such as diabetes, oncological diseases, and cardiovascular diseases and the titer of anti-SARS-CoV-2 antibodies as well as the risk of developing COVID-19. Our results are in contrast with those of studies carried out with a highly dependent population [23,26,27]. The mechanisms by which hypertension increases the risk and severity of COVID-19 are complex and may be linked with other comorbidities. For example, hypertension activates the innate and adaptive immune systems, resulting in the release of cytokines and the strengthening of inflammation [28]. In addition, hypertension (the same as diabetes) exerts unfavorable effects on the gut microbiome, which may be worsened by COVID-19 infection (due to persistent inflammation) [29]. However, there are studies with small groups of subjects, like our study, showing that diabetes and cardiovascular diseases had no relation with the more severe cause of COVID-19 [30]. Further studies are required for a better explanation of related mechanisms in COVID-19 development.

We acknowledge that a small group of subjects is a limitation that prevents general conclusions and strong recommendations. However, the results of our study add supplementary information to the data obtained from other centers [24,30,31] regarding anti-SARS-CoV-2 vaccination and bring new knowledge about the immune response after such vaccination in kidney transplant patients, at the same contributing to further recommendations on how to manage these patients. The main strength of our study is the assessment of COVID-19 morbidity in kidney transplant patients after social isolation due to the pandemic.

In summary, according to our data, a booster dose of the anti-SARS-CoV-2 vaccine stimulates the production of specific antibodies and the cellular immune response. Still, it does not provide effective protection against COVID-19 for most kidney transplant patients. Therefore, patients with poor responses to vaccination should be candidates for a primary prevention strategy, namely avoidance of infection by reducing social contacts and consideration about reducing immunosuppression during the vaccination period, because these patients may develop more severe cases of COVID-19 requiring hospitalization. In addition, the prevalence of the protective efficacy of natural and vaccine-induced immunity against new virus variants [6] and the long-term consequences of the pandemic and the post-pandemic situation on a population scale require further research.

## 5. Conclusions

Almost half of kidney transplant patients may develop a sufficient antibody response after three doses of the anti-SARS-CoV-2 vaccine (BNT162b2). The adequacy of the immune response after vaccination against COVID-19 is associated with kidney function. During the post-pandemic period, no significant differences in COVID-19 cases in relation to serological response to vaccination were observed in kidney transplant patients; however, only patients who did not develop a sufficient antibody response after the COVID-19 vaccine experienced hospitalization due to COVID-19. The anti-SARS-CoV-2 antibody titer relation with the components of cellular immunity (CD3^+^ cells and CD8^+^/CD38^+^ ratio) shows a role of both chains during the response to the COVID-19 vaccine in kidney transplant patients.

## Figures and Tables

**Table 1:** Comparison of demographic and laboratory test results in SARS-CoV-2 seropositive and seronegative groups of kidney transplant recipients before and after the third dose of anti-SARS-CoV-2 vaccination.

		Studied Groups				*p*
		Seronegative (*n* = 19)		Seropositive (*n* = 14)		
		Mean (s)	Median (Min–Max)	Mean (s)	Median (Min–Max)	
Age, years		55 (12)	58 (26–70)	54 (13)	56 (32–82)	0.778
Hemoglobin (mg/L)	Before	133 (7.99)	134.5 (119–145)	138.85 (15.03)	138 (102.0–160.0)	0.271
	After	129.00 (6.68)	129.0 (121–138)	131.22 (17.07)	131 (96.0–156.0)	0.751
eGFR * after vaccination (mL/min/1.73 m^2^)	Before	49.15 (10.69)	45.0 (35.0–71.0)	71.09 (22.06)	65 (43.0–117.0)	0.004
	After	49.43 (9.74)	46.5 (35.0–67.0)	70.64 (21.02)	68.0 (43.0–118)	0.003
Creatinine (mcmol/L)	Before	136.3 (36.28)	146 (81–180)	100.39 (28.82)	90 (68.0–152.0)	0.015
	After	140.0 (37.54)	145 (90–192)	96.44 (28.28)	87.0 (63–139)	0.019
Leucocyte count (×10^9^/L)	Before	7.53 (2.73)	7.55 (4.00–11.5)	7.99 (1.75)	7.8 (5.9–11.3)	0.626
	After	7.39 (2.62)	7.2 (4.4–10.7)	7.59 (1.95)	7.8 (4.8–10.2)	0.861
Lymphocyte count (×10^9^/L)	Before	1.89 (0.77)	1.85 (0.5–3)	2.46 (0.59)	2.1 (1.3–3.1)	0.376
	After	1.53 (0.71)	1.6 (0.4–2.5)	2.17 (0.53)	2.1 (1.5–3.1)	0.058

*—estimated glomerular filtration rate (eGFR).

**Table 2:** The distribution of CD3^+^ and CD8^+^ T cells before and after the third dose of anti-SARS-CoV-2 vaccine in kidney transplant patients.

	Studied Groups					
		Seronegative	Seropositive	Seronegative	Seropositive	
		Mean (S)		Median (Min–Max)		*p*
CD3^+^, %	Before	88.53 (5.18)	87.32 (5.89)	90.35 (80.90–94.22)	86.98 (75-.23–94.93)	0.686
	After	88.35 (1.68)	89.71 (2.17)	88.81 (85.94–89.83)	89.75 (87.13–92.72)	0.339
CD8^+^, %	Before	37.95 (7.66)	33.61 (17.65)	37.91 (25.95–47.60)	28.73 (9.90–68.87)	0.582
	After	33.79 (7.60)	40.54 (16.86)	34.11 (24.48–42.45)	39.37 (23.52–67,40)	0.486

## Data Availability

Not applicable.

## References

[1] Heldman M.R., Limaye A.P. (2021). SARS-CoV-2 vaccines in kidney transplant recipients: Will they be safe and effective and how will we know?. J. Am. Soc. Nephrol..

[2] Aljabr W., Al-Amari A., Abbas B., Karkashan A., Alamri S., Alnamnakani M., Al-Qahtani A. (2022). Evaluation of the Levels of Peripheral CD3 +, CD4 +, and CD8+ T Cells and IgG and IgM Antibodies in COVID-19 Patients at Different Stages of Infection. Microbiol. Spectr..

[3] Castro Dopico X., Ols S., Loré K. (2022). Karlsson Hedestam G.B. Immunity to SARS-CoV-2 induced by infection or vaccination. J. Intern. Med..

[4] Vaiciuniene R., Sitkauskiene B., Bumblyte I.A., Dalinkeviciene E., Ziginskiene E., Bagdonas D., Augliene R., Petruliene K., Bagdziuniene I., Skarupskiene I. (2021). Immune response after sars-cov-2 vaccination in kidney transplant patients. Medicina.

[5] Panizo N., Giménez E., Albert E., Zulaica J., Rodríguez-Moreno A., Rusu L., Giménez-Civera E., Puchades M.J., D’Marco L., Gandía-Salmerón L. (2022). SARS-CoV-2-Spike Antibody and T-Cell Responses Elicited by a Homologous Third mRNA COVID-19 Dose in Hemodialysis and Kidney Transplant Recipients. Microorganisms.

[6] El Karoui K., De Vriese A.S. (2022). COVID-19 in dialysis: Clinical impact, immune response, prevention, and treatment. Kidney Int..

[7] Lee A.R.Y.B., Wong S.Y., Chai L.Y.A., Lee S.C., Lee M.X., Muthiah M.D., Tay S.H., Teo C.B., Tan B.K.J., Chan Y.H. (2022). Efficacy of covid-19 vaccines in immunocompromised patients: Systematic review and meta-analysis. BMJ.

[8] Caillard S., Thaunat O. (2021). COVID-19 vaccination in kidney transplant recipients. Nat. Rev. Nephrol..

[9] Sahin U., Muik A., Vogler I., Derhovanessian E., Kranz L.M., Vormehr M., Quandt J., Bidmon N., Ulges A., Baum A. (2021). BNT162b2 vaccine induces neutralizing antibodies and poly-specific T cells in humans. Nature.

[10] Marlet J., Gatault P., Maakaroun Z., Longuet H., Stefic K., Handala L., Eymieux S., Gyan E., Dartigeas C., Gaudy-Graffin C. (2021). Antibody responses after a third dose of covid-19 vaccine in kidney transplant recipients and patients treated for chronic lymphocytic leukemia. Vaccines.

[11] Kamar N., Abravanel F., Marion O., Couat C., Izopet J., Del Bello A. (2021). Three Doses of an mRNA Covid-19 Vaccine in Solid-Organ Transplant Recipients. N. Engl. J. Med..

[12] Stumpf J., Siepmann T., Lindner T., Karger C., Schwöbel J., Anders L., Faulhaber-Walter R., Schewe J., Martin H., Schirutschke H. (2021). Humoral and cellular immunity to SARS-CoV-2 vaccination in renal transplant versus dialysis patients: A prospective, multicenter observational study using mRNA-1273 or BNT162b2 mRNA vaccine. Lancet Reg. Health –Eur..

[13] Bobcakova A., Barnova M., Vysehradsky R., Petriskova J., Kocan I., Diamant Z., Jesenak M. (2022). Activated CD8+CD38+ Cells Are Associated With Worse Clinical Outcome in Hospitalized COVID-19 Patients. Front. Immunol..

[14] Thomson T., Prendecki M., Gleeson S., Martin P., Spensley K., De Aguiar R.C., Sandhu B., Seneschall C., Gan J., Clarke C.L. (2022). Immune responses following 3rd and 4th doses of heterologous and homologous COVID-19 vaccines in kidney transplant recipients. Eclinicalmedicine.

[15] Reindl-Schwaighofer R., Heinzel A., Mayrdorfer M., Jabbour R., Hofbauer T.M., Merrelaar A., Eder M., Regele F., Doberer K., Spechtl P. (2022). Comparison of SARS-CoV-2 Antibody Response 4 Weeks After Homologous vs Heterologous Third Vaccine Dose in Kidney Transplant Recipients: A Randomized Clinical Trial. JAMA Intern. Med..

[16] Asif S., Frithiof R., Lipcsey M., Kristensen B., Alving K., Hultström M. (2020). Weak anti-SARS-CoV-2 antibody response is associated with mortality in a Swedish cohort of COVID-19 patients in critical care. Crit. Care.

[17] Ganji A., Farahani I., Khansarinejad B., Ghazavi A., Mosayebi G. (2020). Increased expression of CD8 marker on T-cells in COVID-19 patients. Blood Cells Mol. Dis..

[18] Chen Z., John Wherry E. (2020). T cell responses in patients with COVID-19. Nat. Rev. Immunol..

[19] Oberhardt V., Luxenburger H., Kemming J., Schulien I., Ciminski K., Giese S., Csernalabics B., Lang-Meli J., Janowska I., Staniek J. (2021). Rapid and stable mobilization of CD8+ T cells by SARS-CoV-2 mRNA vaccine. Nature.

[20] Sattler A., Schrezenmeier E., Weber U.A., Potekhin A., Bachmann F., Straub-Hohenbleicher H., Budde K., Storz E., Proß V., Bergmann Y. (2021). Impaired humoral and cellular immunity after SARS-CoV-2 BNT162b2 (tozinameran) prime-boost vaccination in kidney transplant recipients. J. Clin. Investig..

[21] Infantino M., Tsalouchos A., Russo E., Laudicina S., Grossi V., Lari B., Benucci M., Stacchini L., Amedei A., Casprini P. (2022). Assessing T-Cell Immunity in Kidney Transplant Recipients with Absent Antibody Production after a 3rd Dose of the mRNA-1273 Vaccine. Int. J. Mol. Sci..

[22] Zhang Z., Mateus J., Coelho C.H., Dan J.M., Moderbacher C.R., Gálvez R.I., Cortes F.H., Grifoni A., Tarke A., Chang J. (2022). Humoral and cellular immune memory to four COVID-19 vaccines. Cell.

[23] Di Castelnuovo A., Bonaccio M., Costanzo S., Gialluisi A., Antinori A., Berselli N., Blandi L., Bruno R., Cauda R., Guaraldi G. (2020). Common cardiovascular risk factors and in-hospital mortality in 3,894 patients with COVID-19: Survival analysis and machine learning-based findings from the multicentre Italian CORIST Study. Nutr. Metab. Cardiovasc. Dis..

[24] Cucchiari D., Egri N., Bodro M., Herrera S., Del Risco-Zevallos J., Casals-Urquiza J., Cofan F., Moreno A., Rovira J., Banon-Maneus E. (2021). Cellular and humoral response after mRNA-1273 SARS-CoV-2 vaccine in kidney transplant recipients. Am. J. Transplant..

[25] Chukwu C.A., Mahmood K., Elmakki S., Gorton J., Kalra P.A., Poulikakos D., Middleton R. (2022). Evaluating the antibody response to SARS-COV-2 vaccination amongst kidney transplant recipients at a single nephrology centre. PLoS ONE.

[26] Mehra M.R., Desai S.S., Kuy S., Henry T.D., Patel A.N. (2020). Cardiovascular Disease, Drug Therapy, and Mortality in Covid-19. N. Engl. J. Med..

[27] Mancusi C., Grassi G., Borghi C., Ferri C., Muiesan M.L., Volpe M., Iaccarino G., SARS-RAS Investigator Group (2021). Clinical Characteristics and Outcomes of Patients with COVID-19 Infection: The Results of the SARS-RAS Study of the Italian Society of Hypertension. High Blood Press Cardiovasc. Prev..

[28] Peng M., He J., Xue Y., Yang X., Liu S., Gong Z. (2021). Role of Hypertension on the Severity of COVID-19: A Review. J. Cardiovasc. Pharmacol..

[29] Sharma R.K., Stevens B.R., Obukhov A.G., Grant M.B., Oudit G.Y., Li Q., Richards E.M., Pepine C.J., Raizada M.K. (2020). ACE2 (Angiotensin-Converting Enzyme 2) in Cardiopulmonary Diseases: Ramifications for the Control of SARS-CoV-2. Hypertension.

[30] Pinchera B., Spirito L., Ferreri L., Rocca R., Celentano G., Buonomo A.R., Foggia M., Scotto R., Federico S., Gentile I. (2022). SARS-CoV-2 in Kidney Transplant Patients: A Real-Life Experience. Front. Med..

[31] Fujieda K., Tanaka A., Kikuchi R., Takai N., Saito S., Yasuda Y., Fujita T., Kato M., Furuhashi K., Maruyama S. (2022). Antibody response to double SARS-CoV-2 mRNA vaccination in Japanese kidney transplant recipients. Sci. Rep..

